# Computational Prediction of Phosphoinositide Binding to Hyperpolarization-Activated Cyclic-Nucleotide Gated Channels

**DOI:** 10.3389/fphys.2022.859087

**Published:** 2022-03-25

**Authors:** Ainara Claveras Cabezudo, Asma Feriel Khoualdi, Nazzareno D’Avanzo

**Affiliations:** ^1^Institute of Pharmacy and Molecular Biotechnology (IPMB), Heidelberg University, Heidelberg, Germany; ^2^Département de Pharmacologie et Physiologie, Université de Montréal, Montréal, QC, Canada

**Keywords:** HCN channel, phosphoinositides, ion channel, lipids, protein-lipid interactions, computational docking, molecular dynamics

## Abstract

Protein-lipid interactions are key regulators of ion channel function. Numerous ion channels, including hyperpolarization-activated cyclic-nucleotide gated (HCN) channels have been shown to be regulated by phosphoinositides (PIPs), with important implications in cardiac and neuronal function. Specifically, PIPs have been shown to enhance HCN activation. Using computational approaches, we aim to identify potential binding sites for HCN1-PIP interactions. Computational docking and coarse-grained simulations indicate that PIP binding to HCN1 channels is not well coordinated, but rather occurs over a broad surface of charged residues primarily in the HCN-domain, S2 and S3 helices that can be loosely organized in 2 or 3 overlapping clusters. Thus, PIP-HCN1 interactions are more resembling of electrostatic interactions that occur in myristoylated alanine-rich C kinase substrate (MARCKS) proteins, than the specifically coordinated interactions that occur in pleckstrin homology domains (PH domains) or ion channels such as inward rectifier potassium (Kir) channels. Our results also indicate that phosphatidylinositol (PI) interactions with HCN1 are even lower affinity, explaining why unphosphorylated PI have no effect on HCN1 activation unlike phosphorylated PIPs.

## Introduction

Hyperpolarization activated cyclic-nucleotide gated (HCN) channels represent the molecular correlate of the currents I_h_ or I_f_, in cardiac and neuronal cells (Brown et al., 1979; Halliwell and Adams, 1982). Four mammalian isoforms exist (HCN1–HCN4), with ∼60% sequence identity. In cardiac conduction tissue, I_h_ serves as the primary initiator for the diastolic depolarization of sinoatrial node (SAN) and atrioventricular node (AVN) action potentials. The sensitivity of HCN channels to cyclic-nucleotides enables I_h_ to adjust to stimulation of the autonomic nervous system. HCN channels are also widely expressed in the central and peripheral nervous systems where they play aid in setting the resting membrane potential, dendritic integration, neuronal pacemaking, and establishing action potential threshold (Pape, 1996) with important roles for learning and memory (Nolan et al., 2003, 2004), pain sensation (Emery et al., 2011), sour taste sensation (Stevens et al., 2001), olfaction (Holderith et al., 2003), and vision (Ivanova and Muller, 2006; Knop et al., 2008).

There is growing evidence that the functions of various ion channels can be modulated by the lipid environment in which they are housed. HCN channels have been shown to be regulated by cholesterol (Barbuti et al., 2004, 2012; Furst and D’Avanzo, 2015), palmitoylation (Itoh et al., 2016), lipopolysaccharides (Zorn-Pauly et al., 2007; Scheruebel et al., 2014; Ebelt et al., 2015) and phospholipids (Pian et al., 2006; Zolles et al., 2006; Ying et al., 2011). Broadly, the most widely examined phospholipids to regulate ion channels are phosphoinositides (PIPs), particularly PI(4,5)P_2_ (Suh and Hille, 2008). PIPs comprise 0.2–1% of the phospholipids in most cell membranes, are found primarily on the cytoplasmic leaflet of the eukaryotic plasmalemma, and are differentially distributed in each sub-cellular membrane (Balla, 2013). PIPs play key roles in the regulation of inward rectifier potassium (Kir) channels (Fan and Makielski, 1997; Huang et al., 1998; D’Avanzo et al., 2010), voltage-gated K^+^ (Kv) channels (Bian et al., 2001; Suh and Hille, 2002; Zhang et al., 2003; Oliver et al., 2004), epithelial Na^+^ channels (Yue et al., 2002), transient receptor potential (TRP) channels (Rohacs and Nilius, 2007), the Na^+^-Ca^2+^ exchanger (Hilgemann and Ball, 1996), P2X receptor channels (Zhao et al., 2007), cyclic-nucleotide gated (CNG) channels (Womack et al., 2000; Zhainazarov et al., 2004; Bright et al., 2007), and HCN channels (Pian et al., 2006; Zolles et al., 2006; Ying et al., 2011) among a long list of other membrane proteins.

Native and heterologously expressed HCN channels undergo “run-down” in excised patches or during prolonged whole-cell recordings. This run-down is associated with a 30–50 mV hyperpolarizing shift in steady-state activation, that cannot be explained by changes in cyclic-nucleotides levels (DiFrancesco et al., 1986; Chen et al., 2001). Intracellular application of PI(4,5)P_2_ reverses the hyperpolarization shift causing run-down, by shifting the voltage-dependence of activation, accelerating channel activation and slowing deactivation (Pian et al., 2006; Zolles et al., 2006; Ying et al., 2011). These effects are conserved between HCN1-4 (Zolles et al., 2006; Ying et al., 2011), implying a common mechanism between isoforms. In addition, the effect of phosphoinositides (PIPs) does not differ between species, with PI(4)P, PI(3,4)P_2_, PI(4,5)P_2_, and PI(3,4,5)P_3_ all inducing the same functional effects. However, phosphatidylinositol (PI) does not have an effect on the voltage-dependence of activation (Pian et al., 2006; Zolles et al., 2006; Ying et al., 2011) indicating an important role of headgroup phosphorylation in modulating HCN function.

Regulation of HCN channels by PIPs may have important functions in cardiac and neuronal physiology. A hyperpolarization shift in I_h_ in the SAN, of the magnitude induced by the depletion of PIPs would be expected to slow the heart rate, similar to the effect of nanomolar concentrations of acetylcholine (DiFrancesco et al., 1989). Pacemaking activity of thalamic intergeniculate leaflet (IGL) neurons also depends on I_h_, with depletion of PI(4,5)P_2_ slowing spontaneous firing, while PI(4,5)P_2_ or PI(4)P enrichment enhanced firing. PIP modulation of I_h_ also alters the firing rating in dopaminergic midbrain neurons of the substantia nigra (DA-SN) (Zolles et al., 2006), with potential impacts on multiple brain functions, including voluntary movement, working memory, emotion and cognition. With the growing understanding of I_h_ in neuronal and cardiac physiology (He et al., 2014), a growing number of processes are being found to depend on the regulation of HCNs by PIPs.

Recent advances in structural biology have enabled the solving of atomic resolution structures of HCN1 (Lee and MacKinnon, 2017, 2019) and HCN4 channels (Saponaro et al., 2021) in several conformations. These structures have enabled several insights in how these channels gate, conduct ions, and bind drugs (Lee et al., 2013; Kasimova et al., 2019; Lee and MacKinnon, 2019; Tanguay et al., 2019; Saponaro et al., 2021). Here, we used computational approaches of docking and coarse-grained (CG) simulations to identify putative binding sites for PIPs in HCN1, to understand their lack of specificity, and to assess why PI does not confer the same effects on HCN activation as its phosphorylated derivatives. These computational experiments were performed on HCN1 with their voltage-sensor domains (VSDs) in conformations that would correspond to the membrane being depolarized (up) and hyperpolarized (down). The high sequence identity within the transmembrane regions of the 4 mammalian HCN isoforms, and the conserved effects of PIPs on their voltage-dependence and kinetics (Zolles et al., 2006; Ying et al., 2011), suggest these binding sites would be conserved among mammalian HCNs.

## Materials and Methods

### System Preparation

The cryo-EM structures the HCN1 channels with VSDs in the up or closed conformation (PDB ID: 5U6O) (Lee and MacKinnon, 2017) and down or activated conformation (PDB ID: 6UQF) (Lee and MacKinnon, 2019) were used for these studies. Missing loops were generated in ICM-Pro (Cardozo et al., 1995) (Molsoft LLC, La Jolla), and the structures were then processed through CHARMM-GUI PDB Reader (Jo et al., 2008) to assist with repairing any missing atoms, repair any improper bond lengths or angles. For each modeled structure, the pKa value of each reside was calculated with the PROPKA server (Olsson and Søndergaard, 2011), and all residues were assigned their standard protonation state at pH 7 accordingly. The protein was then oriented appropriately for molecular dynamics simulations using the Orientation of Proteins in Membranes (OPM) webserver (Lomize et al., 2006).

### Computational Docking

To facilitate docking, only the head groups for each of the seven PIPs and PI were used in our docking calculations, with the PI/PIPs truncated at the first carbon atom of glycerol moiety, as previously done for examining PIP binding to Kir channels (D’Avanzo et al., 2013; Lee et al., 2013). The HCN1 channels with VSDs in the up or down conformations and ligands were prepared for docking calculations, including protonation, assigning Gasteiger charge, merging of non-polar hydrogen to the bonded carbon atom, assigning the torsion trees (for each ligand) and assigning atom type using AutodockTools4.2.6 (ADT4) (Morris et al., 1998). Grid parameter files and grid maps were generated by AutoGrid 4.2 within ADT with a grid spacing of 0.556 Å. The search area used for docking calculations were limited to the region in which the lipid head groups can reach with the lipid tail still embedded in the membrane (i.e., the intracellular portion of the transmembrane helices, the entire HCN domain, the C-linker and the upper region of the CNBD). While the membrane is not present in the docking simulations, its potential location with respect to the protein can be approximated based the output from the OPM server used in preparation of the proteins. Each ligand was independently docked 500 times using Lamarckian GA docking algorithm (Morris et al., 2009) using default parameters except the maximum number of energy evaluations was raised to 25,000,000. To account for the four-fold symmetry of HCNs, docked ligand poses were rotated into the same quadrant and automatically accepted and clustered by (i) the orientation of functional groups, and (ii) position using the MultiCluster software as previously described (Tanguay et al., 2019). Ligands were simplified to a representative vector generated by calculating centroids of atoms from three different groups of atoms within the molecule: (i) the glycerol carbon, (ii) the inositol ring and (iii) one of the phosphate groups at the 3, 4, or 5 position. Using this software, poses are accepted only if the glycerol carbon was parallel or facing toward the membrane bilayer, and clustered if centroids were within 15 Å of one another. Residues within 4 Å of the ligand are then automatically identified by the software.

### Coarse-Grained Molecular Dynamics Simulations

PDBs of the HCN1 with VSDs in the up or down conformation prepared as above were then converted to CG representations using the CHARMM-GUI Martini Maker webserver (Jo et al., 2008; Qi et al., 2015). The HCN1 channel was embedded into a DOPC membrane containing 8–9 (3%) randomly placed PIP3 molecules [POP3 in the Martini force-field (FF)] or PI molecules (POPI in the Martini FF) in the inner leaflet. The systems were solvated with polarizable water molecules (Yesylevskyy et al., 2010) and neutralized in 150 mM NaCl.

All CG simulations were performed using GROMACS version 2018.6 (Abraham et al., 2015) using the Martini2.2p polarizable FF (de Jong et al., 2013). Periodic boundary conditions were applied, and a time step of 20 fs was used in all simulations. The temperature was maintained at 310.15 K using a velocity rescale coupling scheme and the pressure at 1 bar using a Berendsen barostat for equilibration steps (Berendsen et al., 1984) and the Parrinello-Rahman barostat (Parrinello and Rahman, 1981) was used for production steps. For both the temperature and pressure, a coupling constant of 4 ps was used. In all simulations, the reaction field coulomb type was used with a switching function from 0.0 to 1.1 nm, and the van der Waals interactions were cutoff at 1.1 nm. For simulations of HCN1 with VSDs in the down (activated) conformation, a -400 mV electric field was applied in the direction of the *z*-axis. The LINCS algorithm (Hess et al., 1997) was used to constrain covalent bonds to their equilibrium values. Production simulations were run for 30 μs for all 4 systems (HCN1 up or down with PIP3 or PI) using resource allocations granted through ComputeCanada and CalculQuebec. Root-mean squared deviations (RMSD) of HCN1 channels were calculated by a GROMACS tool, g_rms. Protein–lipid interactions were identified using PyLipID software (Song et al., 2022) with lipid headgroups considered to be interacting with the HCN1 channel residues when within 0.5 and 0.8 nm. Clustering of binding sites was performed by analysis of interactions with the lipid head-group within 0.8 nm. Lipid density distribution for POP3 and POPI were calculated using the ProLint server (Sejdiu and Tieleman, 2021).

## Results

### Identification of HCN1 Residues That Interact With Phosphoinositides by Computational Docking

**FIGURE 1:**
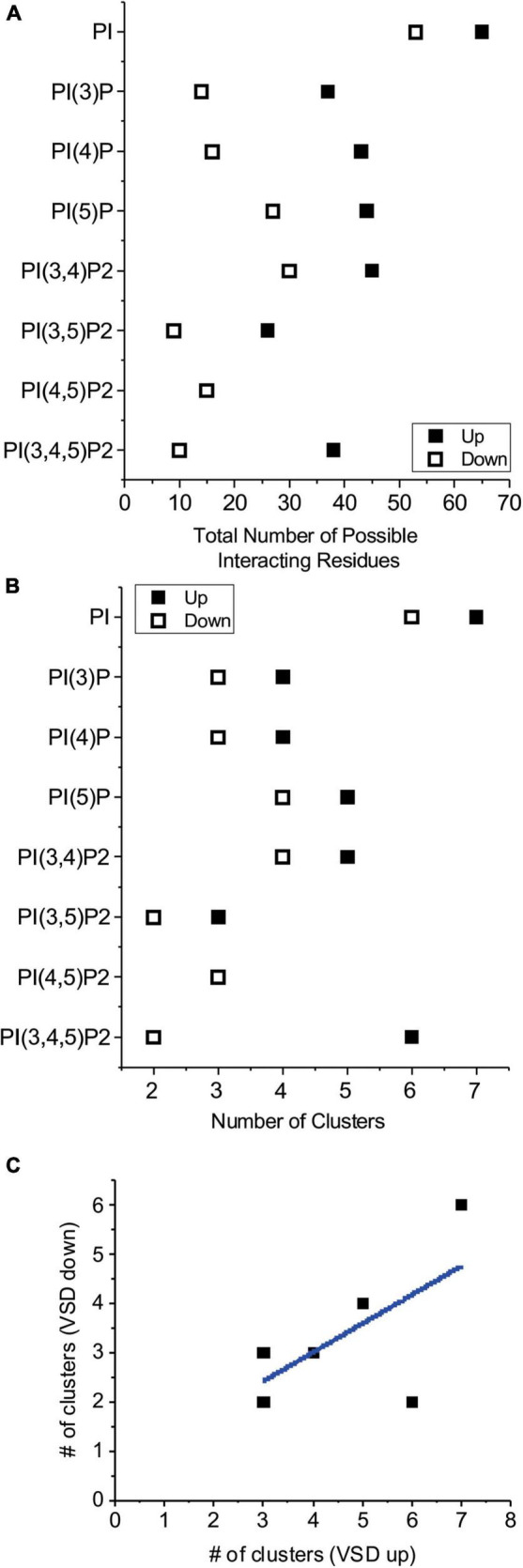
Number of residues that interact with PIPs by computational docking. **(A)** The total number of residues within 4 Å of any part of the listed ligand in all accepted poses were summed for HCN1 channels with voltage-sensor in the up (◼) or down (□) conformations. These results suggest phosphorylated PIPs are more readily coordinated in fewer regions of the channel compared to unphosphorylated PI. In all cases, PI and PIPs bind to fewer residues in when the voltage-sensor is in the down conformation compared to the up conformation. **(B)** The number of clusters for each lipid ligand for HCN1 channels with voltage-sensor in the up (◼) or down (□) conformations. Generally, lipid poses are more localized (fewer clusters) when the voltage-sensor is in the down (activated) conformation, than thin the up (deactivated) conformation. **(C)** The number of clusters in the down state is correlated to the number of clusters observed in the up state (Pearson’s *r* = 0.63; R^2^ COD = 0.40).

To gain insight into the possible sites where PIPs interact with HCN channels, and why these channels can be similarly activated by all PIP species, except for unphosphorylated PI, we used computational ligand docking experiments on atomic structures of HCN1 with their VSDs in the deactivated depolarized conformation (up) (PDB: 5U6O) and the activated hyperpolarized conformation (down) (PDB: 6UQF). Since the acyl-chain properties are not the primary driver for channel activation we used only the headgroups of the PIP ligands truncated with the C1 carbon still covalently linked to the P1 phosphate for each ligand, as previously done (D’Avanzo et al., 2013; Lee et al., 2013). Each ligand was independently docked to each HCN1 conformation 500 times. Docking poses (PIP/PI bound HCN1 structures) were analyzed for correct orientation, with poses in which the “tails” (glycerol C1 carbon) were oriented facing away from the expected plane of the membrane removed. Therefore, poses were “accepted” if the C1 carbon was parallel or facing the direction of the membrane bilayer/transmembrane helices. Accepted poses were automatically clustered using our previous described Multicluster software (Tanguay et al., 2019) with a 15 Å cut-off. Prior to assessing the specifics of each cluster, it is notable that the total number of residues that interact with the ligands are fewer for PIPs than for PI, in both the up and down conformations (Figure 1A). Furthermore, it is also notable that each ligand interacts with fewer residues in HCN1 with their VSDs in the down (hyperpolarized activated) conformation than in the up (depolarized deactivated) (Figure 1A). In the up conformation, PIPs interact with 15–45 residues depending on the lipid species, while PI interacts with 65 residues (17–52% and 76%, respectively of the 86 residues per subunit that comprise the lipid accessible surface of the intracellular leaflet). In the down conformation, PIPs interact with 9–30 residues, while PI interacts with 53 residues (9.5–32% and 56%, respectively of the 94 residues that comprise the lipid accessible surface of the intracellular leaflet). Lastly, we also observe that generally fewer clusters are identified for PIPs than PI, and again fewer clusters for each ligand in the down conformation than in the up conformation (Figure 1B). These pseudo-entropic results suggest that PI may be less easily coordinated by the HCN1 channel than its phosphorylated counterparts, and that PIP interactions occur more readily or are more favorable upon membrane hyperpolarization when the VSD moves in the down conformation. This is supported by the correlation between the number of clusters in the up and down conformations (Figure 1C).

**FIGURE 2:**
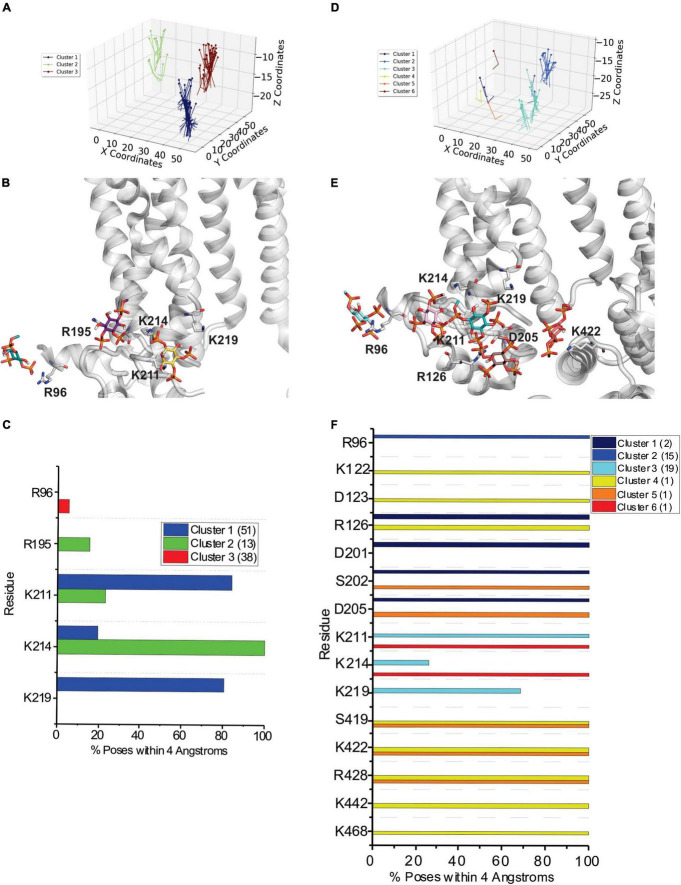
Docking of PI(4,5)P_2_ and PI(3,4,5)P_3_ to HCN1 channels with VSDs in the up (closed) conformation. **(A)** The results from 500 attempts to dock PI(4,5)P_2_ headgroups to HCN1 (PDB: 5U6O). Poses were accepted based on orientation and clusters were identified in an automated manner using MultiCluster software previously described (Tanguay et al., 2019). **(B)** Binding sites of the most representative poses for each cluster **(C)** The frequency of residues within 4 Å of the PI(4,5)P_2_ ligand for each pose was assessed and is indicated as a percentage of the accepted poses. **(D)** The results from 500 attempts to dock PI(3,4,5)P_3_ headgroups to HCN1. **(E)** Binding sites of the most representative poses for each cluster **(F)** The frequency of residues within 4 Å of the PI(3,4,5)P_3_ ligand for each pose was assessed and is indicated as a percentage of the accepted poses.

**FIGURE 3:**
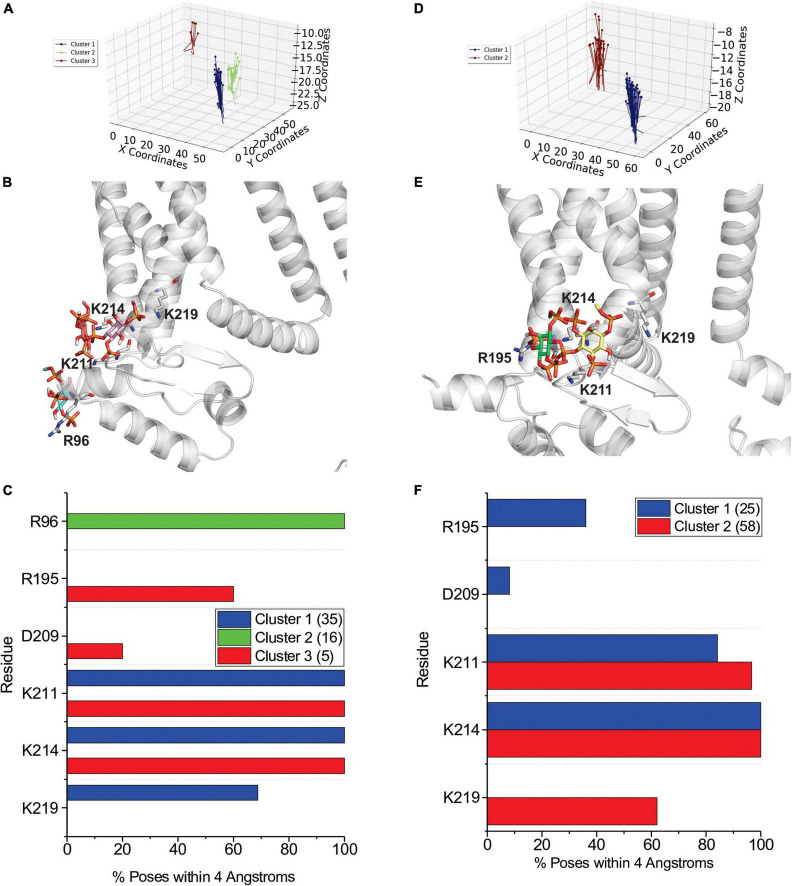
Docking of PI(4,5)P_2_ and PI(3,4,5)P_3_ to HCN1 channels with VSDs in the down (activated) conformation. **(A)** The results from 500 attempts to dock PI(4,5)P_2_ headgroups to HCN1 (PDB: 6UQF). Poses were accepted based on orientation and clusters were identified in an automated manner using MultiCluster software previously described (Tanguay et al., 2019). **(B)** Binding sites of the most representative poses for each cluster **(C)** The frequency of residues within 4 Å of the PI(4,5)P_2_ ligand for each pose was assessed and is indicated as a percentage of the accepted poses. **(D)** The results from 500 attempts to dock PI(3,4,5)P_3_ headgroups to HCN1. **(E)** Binding sites of the most representative poses for each cluster **(F)** The frequency of residues within 4 Å of the PI(3,4,5)P_3_ ligand for each pose was assessed and is indicated as a percentage of the accepted poses.

A broad examination of our docking results indicates that PIP binding to HCN1 channels does not appear to be specifically coordinated by key residues that form a “binding pocket” as it is for some other channels such as Kir channels (Hansen et al., 2011; D’Avanzo et al., 2013; Lee et al., 2013; Duncan et al., 2020; Niu et al., 2020). Instead, PIP binding appears smeared over a larger surface, with some clusters sharing a number of residues with a neighboring cluster (Figures 2, 3 and Supplementary Figures 1–12). For clarity, we have limited our presentation to focus on the polar residues within putative binding sites, since electrostatic interactions and hydrogen bonding are expected to be the primary stabilizing interactions. Effectively, our docking analysis indicates that PIP interactions with HCN1 channels involve two regions of the channel when VSDS are in either the up or down conformation. Firstly, PIPs can interact with charged or polar residues on the outer edge of the channel-membrane interface in the HCN domain. Here PIPs primarily interact with R96, but frequently T99, S100, K108, K122, R126, and K128, though these latter interactions only occur in the up conformation. A second region of PIP binding involves residues at the bottom of the S2 helix particularly R195, and S3 helix K211, K214, and K219. Some clusters extend deeper to include the S4 residues including R273 and R276, however, clusters that include these residues are most often poorly occupied (i.e., contain a low number of accepted poses). We also observe several clusters that interact with numerous residues on the C-linker and CNBD, including residues K422, R428, K442, and K468. We have included these clusters for completeness, however, it is likely that interactions at these positions are not the primary sites of PIP interactions, since the effects of PIPs on HCN activation have been observed in HCN channels lacking their CNBD (Suh and Hille, 2008). Therefore, we would expect the most important PIP binding sites that affect HCN activation to reside primarily in the HCN domain, transmembrane helices and/or intracellular loops.

### PI and PIP3 Interactions With HCN1 Assessed by Coarse-Grained Simulations

**FIGURE 4:**
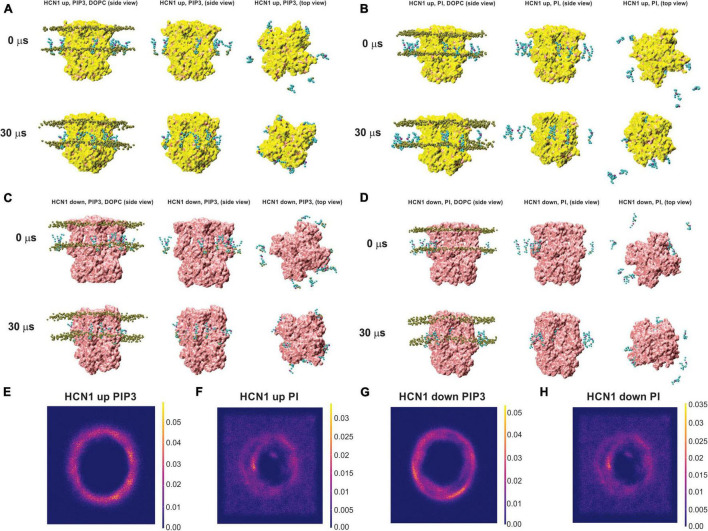
Coarse-grained (CG) simulations of POP3 and PI for HCN1 with activated and deactivated VSDs. Snapshots of HCN1 (yellow) with the VSD in the up (closed) or down (activated) conformations embedded in a DOPC membrane doped with PIP3 (POP3 in the Martini FF) **(A,C)** or POPI **(B,D)** at the onset and after 30 μs of CG simulations. For orientation, phosphate headgroups of DOPC are shown in brown on the left of each panel, but are removed in other panels for clarity.

As a second approach to identify putative PIP binding sites, we performed CG simulations of HCN1 channels with VSDs in the up or closed conformation (PDB ID: 5U6O) (Lee and MacKinnon, 2017) and down or activated conformation (PDB ID: 6UQF) (Lee and MacKinnon, 2019). Channels were embedded in a DOPC membrane randomly doped with 8–9 molecules of PIP3 (POP3) or POPI in the intracellular leaflet and were run for ∼30 μs following system equilibration (Figure 4). For each system, the protein stabilized within the first 5–8 μs, with less than a 1 Å change for the remainder of the simulations (Supplementary Figure 14). Qualitatively, we observe that by the end of the 30 μs simulations, all PIP3 molecules reside are bound to the channel for HCN1 with VSDs in either the up and down conformation (Figures 4A,C). However, several of the PI molecules remain unbound for both systems (Figures 4B,C). Quantitatively, over the trajectory of the simulations, we observe a greater lipid density surrounding the protein for systems containing PIP3 (Figures 4E,G) compared to systems containing PI (Figures 4F,H). Moreover, rather than observing 4 or 8 punctuated locations of elevated lipid (PI or PIP3) density, we see the lipid density is diffused along the in an annulus surrounding the protein.

**FIGURE 5:**
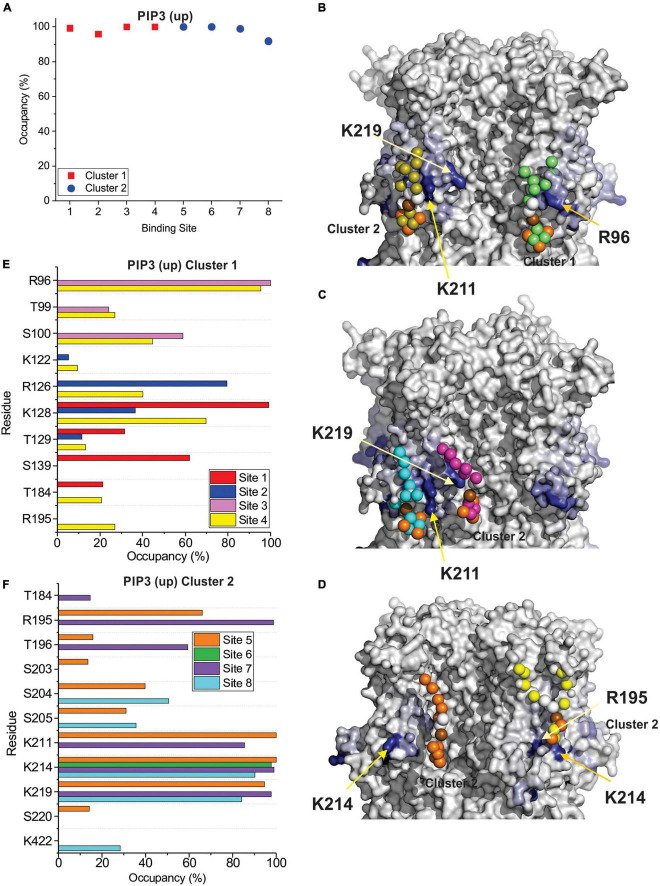
PIP3 binding to HCN1 examined with up (deactivated) VSDs examined by coarse-grained simulations. **(A)** Occupancy (percentage of frames) of each lipid binding site in the HCN1 tetramer identified and quantified using PyLipid software. Binding sites were clustered according to the commonality of residues involved in binding. Two binding sites for PIP3 were identified. **(B–D)** Snapshots of PIP3 lipids (colored balls) bound to HCN1 (surface representation). Residues are colored according to occupancy throughout the trajectory from white (0%) to deep blue (100%). Notably, the occupancy of lipid binding is not confined to a few residues, but smeared over a relatively large surface involving numerous residues. **(E,F)** Occupancy of PIP3-HCN1 interactions for each polar residue. In common with computational docking experiments, binding largely occurred with residues in **(E)** (cluster 1) the HCN domain (including R96, R126, K128) and **(F)** in the S2 (R195) and S3 helices (K211, K214, and K219) (cluster 2).

**FIGURE 6:**
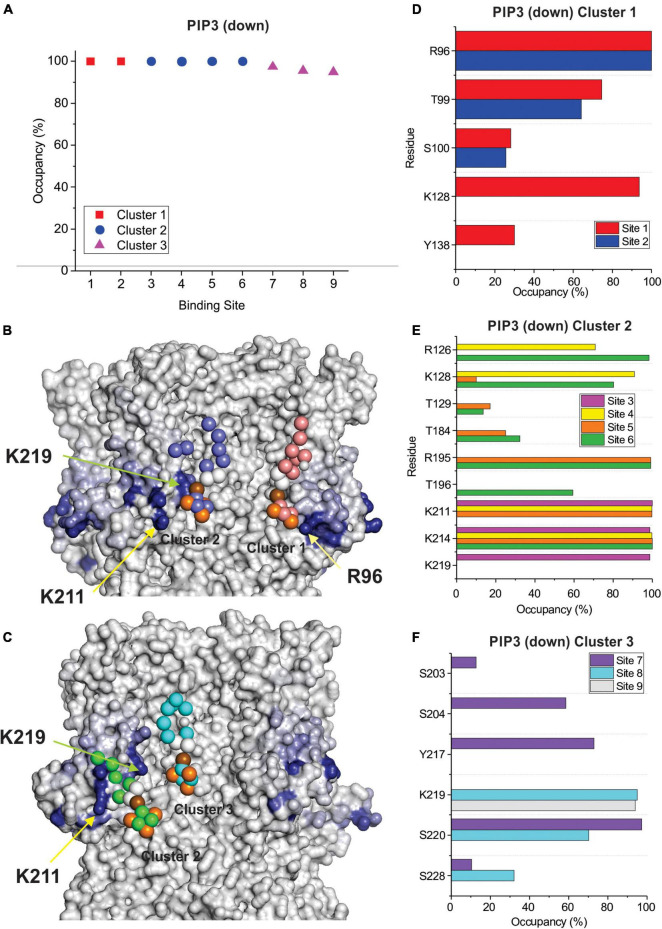
PIP3 binding to HCN1 with down (activated) VSDs examined by coarse-grained simulations. **(A)** Occupancy (percentage of frames) of each lipid binding site in the HCN1 tetramer identified and quantified using PyLipid software. Binding sites were clustered according to the commonality of residues involved in binding. Three binding sites for PIP3 were identified. **(B,C)** Snapshots of PIP3 lipids (colored balls) bound to HCN1 (surface representation). Residues are colored according to occupancy throughout the trajectory from white (0%) to deep blue (100%). Notably, the occupancy of lipid binding is not confined to a few residues, but smeared over a relatively large surface involving numerous residues. **(D–F)** Occupancy of PIP3-HCN1 interactions for each polar residue. In common with computational docking experiments and CG simulations of HCN1 up, binding largely occurred with residues in **(D)** (cluster 1) the HCN domain (including R96, R126, K128) and **(F)** in the S2 (R195) and S3 helices (K211, K214, and K219) (cluster 2) and somewhat deeper toward the pore (cluster 3).

In-depth analysis of lipid binding from our CG simulations largely corroborates our findings from our docking analysis. Similar to our docking results, we observe two broad clusters of PIP2 binding sites, that do not tightly co-ordinate the lipid headgroups as observed for example in Kir channels (Hansen et al., 2011; D’Avanzo et al., 2013; Lee et al., 2013; Duncan et al., 2020; Niu et al., 2020), but rather widely interact with a series of positively charged residues along the outer surface of the channel. The first cluster primarily involves residues in the HCN domain including R96, T99, R126, and K128 (Figures 5, 6). The second cluster primarily involves interactions with residues R195 at the bottom of the S2 helix, and K211, K214, and K219 in the S3, though other polar residues in the region are sometimes involved such as T196 and S204 (Figures 5, 6). For HCN1 with the activated down VSD, a neighboring third cluster is also observed, that is somewhat an extension of cluster 2, interacting with K219 but residing a little deeper toward the central pore and interacting with S220 and S228. We have separated these clusters because in the trajectory binding at both positions at the same time could be observed in some subunits (e.g., Figure 6C). Notably, there are overlapping residues between clusters. For example, PIP2 sometimes binds to K128 or R195 in some cases on the side facing the HCN domain residues such as R96, and in other subunits on the opposite face to interact with K211.

**FIGURE 7:**
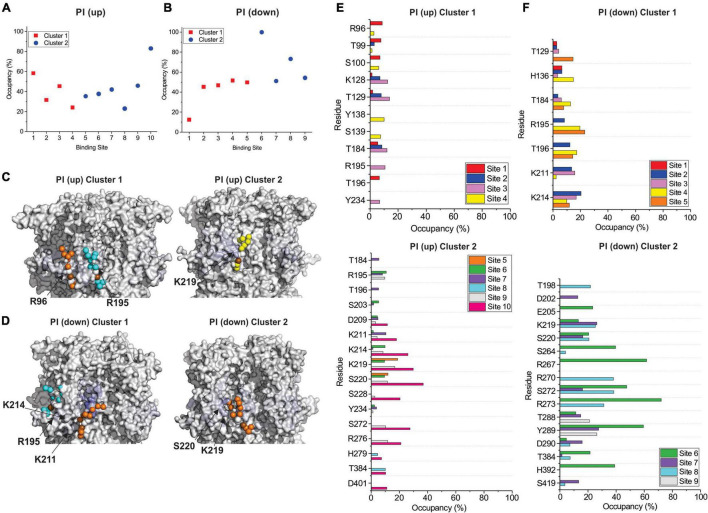
Coarse-grained simulations identify weak binding of POPI to HCN1 with up (closed) and down (activated) VSDs. Occupancy (percentage of frames) of each lipid binding site in the HCN1 tetramer identified and quantified using PyLipid software for HCN1 up **(A)** and HCN1 down **(B)**. Binding sites were clustered according to the commonality of residues involved in binding with 2 low occupancy POPI binding sites identified. **(C)** Snapshots of POPI lipids (colored balls) bound to HCN1 up **(C)** and HCN1 down **(D)** (surface representation). Residues are colored according to occupancy throughout the trajectory from white (0%) to deep blue (100%) as in Figures 5, 6. Notably, the occupancy of lipid binding is not confined to a few residues, but smeared over a relatively large surface involving numerous residues. **(E,F)** Occupancy of PI-HCN1 interactions for each polar residue for HCN1 up **(E)** and HCN1 down **(F)** systems. In common with computational docking experiments, PI interactions occur over a larger number of residues than PIPs. With the VSD in the up state, PI interacts with the HCN domain (including R96, R126, K128) (up cluster 1) and in the S2 (R195) and S3 helices (K211, K214, and K219) (up cluster 2). However, with the VSD in the down state, binding occurs mostly at the S2 and S3 (down cluster 1), and somewhat deeper toward the pore (down cluster 2). And not near the HCN domain. The low occupancy of all POPI interactions indicates weaker binding of PI compared to PIPs.

By examining PI binding with HCN1 channels, we also get a clearer picture that they likely fail to enhance HCN1 activation in part due to their low affinity interactions. Notably, the occupancy of most binding sites in either HCN1 with VSDs either in the up or down conformations are below 60% (Figures 7A,B) compared to PIP3 which are all above 90% occupied (Figures 5A, 6A). Visually this is evident by the lighter blue coloring of residues in Figures 7C,D compared to Figures 5B–D or Figures 6B,C. Again, similar to our docking results, we also observe short-lived interactions over a greater number of residues for PI compared to PIP3 (Figures 7E,F), indicating weak binding with any particular set of residues. Interestingly, with the VSD down, PI appears to most frequently sample the deeper site (equivalent to PIP3 cluster 3 in Figure 6), while the outer HCN-domain site (e.g., in the region of R96) is poorly sampled. Interestingly, when the voltage-sensor is up, this site is occupied by PIP3 on all four subunits through the majority of the trajectory (Figures 5A,E), whereas this site is occupied in only two subunits when the VSD is down (Figures 6A,D). These results suggest that PI and PIP binding may be state dependent, at least at the HCN domain.

## Discussion

Electrophysiology recordings of native I_h_ and heterologously expressed HCN channels have established that phosphoinositides (PIPs) induce a depolarizing shift in steady-state activation, accelerate channel activation and slow deactivation in all four HCN isoforms (Pian et al., 2006; Zolles et al., 2006; Ying et al., 2011). Phosphatidylinositol (PI), on the other hand, does not have an effect on HCNs. However, our understanding of the molecular details of PIP-HCN interactions is currently limited.

The molecular basis for PIP-protein interactions can arise from a combination of specific coordinated interactions and non-specific electrostatic interactions. On one extreme, myristoylated alanine-rich C kinase substrate (MARCKS), a 331-residue natively unfolded protein (Tapp et al., 2005) contains a “basic effector domain” of 13 basic amino acids that confers a strong local positive electrostatic potential to the protein. Unphosphorylated MARCKS can bind the acidic headgroups of PIP2 and PIP3 through non-specific electrostatic interactions and sequester these lipids laterally across the membrane (Wang et al., 2002). Specificity for interacting with multi-phosphorylated PIPs over mono-phosphorylated PIPs likely results from the multiple negative charges in the lipid headgroup, and because PI(4,5)P_2_ is generally the most abundant multi-phosphorylated lipid in the plasma membrane. On the other end of the spectrum, pleckstrin homology domains (PH domains) are found in numerous cytoplasmic proteins. PH-domains all appear to have a common structure consisting of two perpendicular anti-parallel beta sheets, followed by a C-terminal amphipathic helix, forming a pocket that contains several basic amino acids that are positioned and oriented in a manner that specifically enable the co-ordination of a PIP. Several basic residues co-ordinate the phosphates around the inositol ring, while other hydrogen bond interactions between protein and lipid occur *via* uncharged residues to stabilize the lipid (Lemmon, 2003). Specificity for various PIP species in different PH-domains arises from the differing co-ordination patterns of the headgroup phosphates resulting from the specific location and orientation of the basic residues within the binding pocket.

In order to assess the molecular details of PIP binding in HCN channels, we utilized computational docking and CG molecular dynamics simulations. Since the effect of PIPs does not differ between species (Pian et al., 2006; Zolles et al., 2006; Ying et al., 2011), our approach was to look for commonalities between all seven PIP species. Moreover, since lipid binding, like any ligand, can be state-dependent, we examined binding using atomic structures of HCN1 with the VSD domain in the activated (down) and deactivated (up) conformations. Our computational results suggest that PIP-HCN interactions do not occur as the result of well-coordinated and specific interactions as seen in other channels such as Kirs (Hansen et al., 2011; D’Avanzo et al., 2013; Lee et al., 2013; Duncan et al., 2020; Niu et al., 2020). Instead, PIPs appear to bind HCN channels across a smeared surface of charged and polar residues that can be loosely clustered. The most common residues of interactions identified in both docking and CG approaches include residues in the HCN domain (cluster 1) and at the bottom of the S2 and S3 helices (cluster 2). Common to both approaches,—which notably use different functions for their energy calculations—in the HCN domain PIPs primarily interact with R96, but frequently T99, S100, R126, and K128. The most common residues involved in the second cluster of PIP binding are R195 at the bottom of the S2 helix and K211, K214, and K219 residues in the S3 helix. Some binding extends this binding region deeper toward the pore toward residues S220 and S228. However, binding at these residues are less well occupied (i.e., contain a low number of accepted poses in docking or reduced occupancy in CG simulations). Thus, PIP binding in mammalian HCN channels appears to be MARCKS-like involving non-specific electrostatic interactions within a broad region of the channel, rather than well-oriented and coordinated as in PH-domains and Kir channels. This is consistent with the general lack of specificity in response to various PIP species (Pian et al., 2006; Zolles et al., 2006; Ying et al., 2011). Our results also address why phosphatidylinositol (PI) does not have an effect on the activation of HCNs. In both computational docking and CG simulations, we observe PI interacting with a larger number of residues than PIPs in either HCN1 conformation. Furthermore, the occupancy of each PI-HCN1 interaction is low, indicating that these interactions are short-lived (i.e., low affinity).

Our results also hint at state-dependence to PIP binding. In-line with this, in our computational docking experiments, with the exception of PI(4,5)P_2_, we observe a reduction in the number of accepted poses for PIPs in the up state (8–13%) compared to the down state (17–24%). We also observe that accepted poses in the down conformation form fewer clusters and interact with fewer residues than the accepted poses of HCN1 with VSDs in the up state (Figure 1). These differences may arise from the conformational changes of that occur when the VSDs move downward. The HCNa helix of the HCN-domain rotates R96, T99, and S100 residues away from the plane of the plasma membrane, and changes in the S2 and S3 move K211 and K214 closer to R195 in the down state compared to the up state (Supplementary Figure 13). Together, these results suggest that binding of PIPs may be entropically more favorable in the open (VSD down) state, leading to its stabilization. Interestingly, we observe the reverse trend for PI, with 26% of poses accepted with the VSD in the up state, compared to 6% of poses accepted in the down state, suggesting the PI binding may be entropically more favorable in closed channel. Although, it is unclear since there remain fewer clusters and interacting residues in the channels with the open (down) VSD. For both computational methods, we observe more frequent interactions with the HCN domain in closed HCN1 up state than we do in the down state. On the other hand, interactions with transmembrane residues more proximal to the pore than K219 appear to occur more frequently in the HCN1 down state. Thus, while our results are suggestive, more detailed electrophysiology experiments will be needed in order to better address the presence of state-dependent PIP binding. Important to note, the HCN1 down state used in this study represents the channel in an uncoupled conformation, since the pore remains closed even though the VSD has moved downward. Further studies of changes to a fully open channel, as recently observed for HCN4 (Saponaro et al., 2021), will help further elucidate whether there is state-dependent PIP binding in HCNs.

Binding of PIPs at more than one site in HCN channels is consistent with predictions from sea-urchin spIH channels (Flynn and Zagotta, 2011), although the predicted residues of interaction are different. However, this is not entirely unexpected, since PIPs have dual and contrasting effects on spIH channels, with potentiation of voltage-dependent gating, but inhibition of ligand gating. This inhibition of ligand gating does not appear to occur in mammalian HCN channels, since PIP2 does not alter the cAMP-mediated shift in HCN gating nor maximal conductance (Gmax) (Pian et al., 2006; Zolles et al., 2006). Moreover, the effects of PIP2 on the HCN2 voltage-dependence remained even in HCN2ΔCNBD and R591E channels which are unaffected by cAMP (Zolles et al., 2006). The effects on ligand-dependent gating are proposed to involve residues R478 (Q408 in hHCN1) and K482 (K412 in hHCN1) on the spIH C-linker to stabilize the closed state. Since we do not observe binding at these residues in either our docking or our simulations, and since PIPs appear to stabilize the open state of mammalian HCNs, it is likely that the Q408 residue may reduce the favorability of binding at this site compared to in spIH channels, to prevent effects on ligand gating. However, we did observe binding to other residues in the C-linker (e.g., K422 and R428) in the up (closed) conformation with both computational methods, even though the occupancy of these interactions was low. Thus, while binding at the C-linker may be possible in mammalian channels, it appears easily overcome and to have a less dominant effect on their function. On the other hand, the binding site that alters voltage-dependent gating in spIH channels was also proposed to involve the transmembrane regions (Flynn and Zagotta, 2011) though specific residues were not identified. Here we propose charged and polar residues in the HCN domain, S2 and S3 that are largely conserved amongst mammalian HCNs and spIH. The only exceptions are that the equivalent residues to K211 in HCN2 and HCN4 are glutamate and glutamine respectively, and HCN1 K214 is an alanine in spIH. However, given the distribution of charges in this region, and the more MARCKS-like binding, these single amino-acid differences are likely insufficient to disrupt the overall effect of PIP on voltage-dependent activation, since nearby charges would be readily available.

Taken together, our data suggests that PIPs are a relatively low-affinity modulator of HCN channels that interact over a diffuse region, rather than in a well-coordinated “binding pocket.” It has been suggested that low PIP–binding affinities in ion channels may be physiologically important because the slow binding and unbinding may permit a more substantial range of modification by factors that alter PIP levels over slow time-scales (seconds) than high affinity interactions that may be better suited for proteins that require more immediate and drastic activation or antagonism by a lipid regulator (Suh and Hille, 2008).

## Data Availability Statement

The raw data supporting the conclusions of this article will be made available by the authors, without undue reservation.

## Author Contributions

AC, AF, and ND’A performed and analyzed the experiments. AC and ND’A prepared the manuscript. All authors contributed to the article and approved the submitted version.

## Conflict of Interest

The authors declare that the research was conducted in the absence of any commercial or financial relationships that could be construed as a potential conflict of interest.

## Publisher’s Note

All claims expressed in this article are solely those of the authors and do not necessarily represent those of their affiliated organizations, or those of the publisher, the editors and the reviewers. Any product that may be evaluated in this article, or claim that may be made by its manufacturer, is not guaranteed or endorsed by the publisher.
