# Supplementary material for: Computational Prediction of Phosphoinositide Binding to Hyperpolarization-Activated Cyclic-Nucleotide Gated Channels
Source: Front Physiol. 2022 Mar 25;13:859087. doi: 10.3389/fphys.2022.859087 (PMC8990809; doi:10.3389/fphys.2022.859087)
Supplement: Supplementary file 1 [file Data_Sheet_1.docx]

Computational Prediction of Phosphoinositide Binding to Hyperpolarization-activated Cyclic-nucleotide gated (HCN1) Channels

Ainara Claveras Cabezudo^1,2^, Asma Feriel Khoualdi^2^, Nazzareno D'Avanzo^2^

^1^Heidelberg University, Institute of Pharmacy and Molecular Biotechnology (IPMB), Heidelberg, Germany.

^2^Université de Montréal, Département de pharmacologie et physiologie, Montréal, Québec, Canada

***Correspondence:** Nazzareno D’Avanzo nazzareno.d.avanzo@umontreal.ca

Keywords: HCN channel, phosphoinositides, ion channel, lipids, protein-lipid interactions

Supplementary Material

# Supplementary Figures and Tables

**
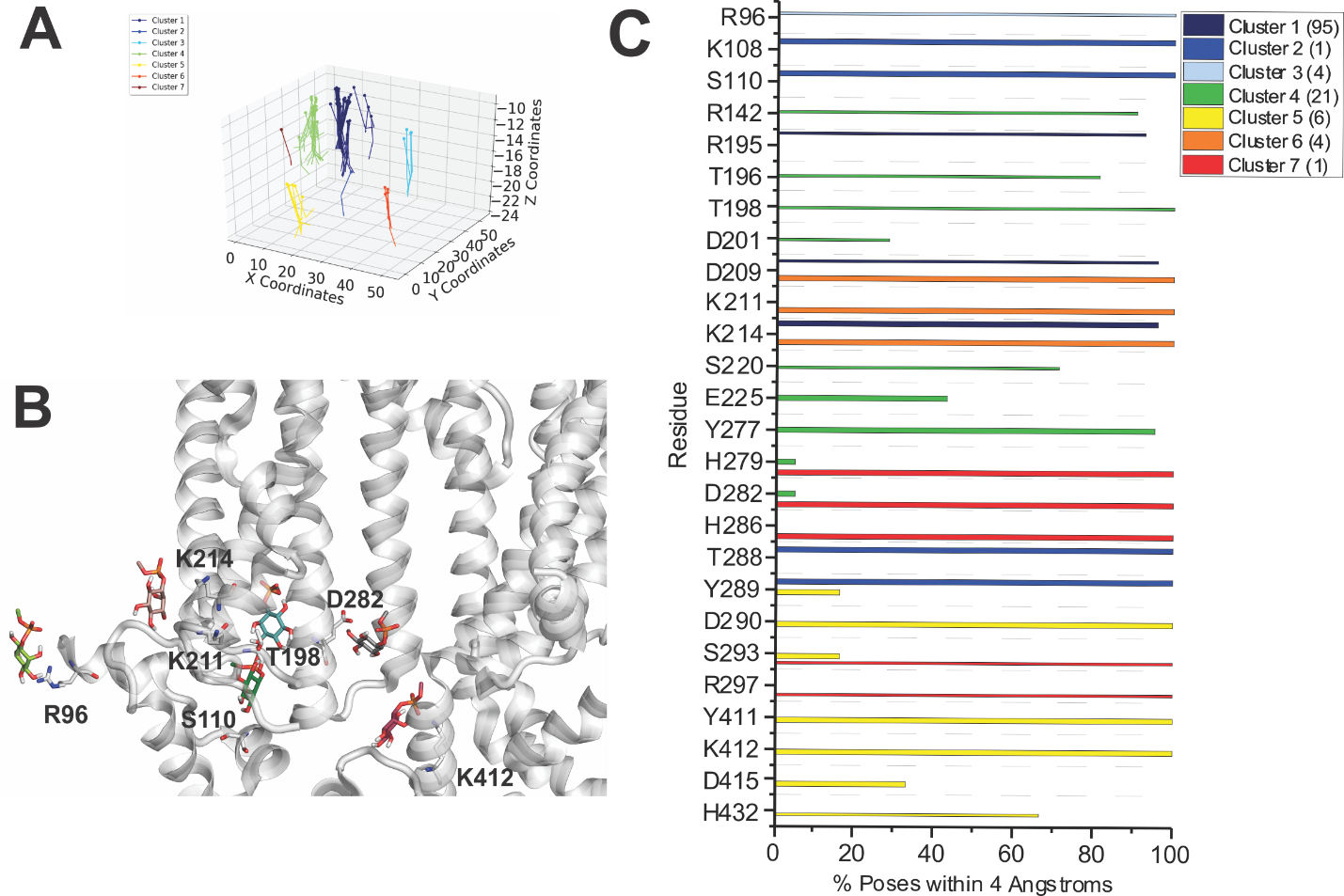
**

**Supp. Fig. S1**. **Docking of PI to HCN1 channels with VSDs in the up (closed) conformation. (A)** The results from 500 attempts to dock PI headgroups to HCN1 (PDB: 5U6O). Poses were accepted based on orientation and clusters were identified in an automated manner using MultiCluster software previously described (Tanguay et al., 2019). **(B)** Binding sites of the most representative poses for each cluster **(C)** The frequency of residues within 4 Å of the ligand for each pose was assessed and is indicated as a percentage of the accepted poses.

**
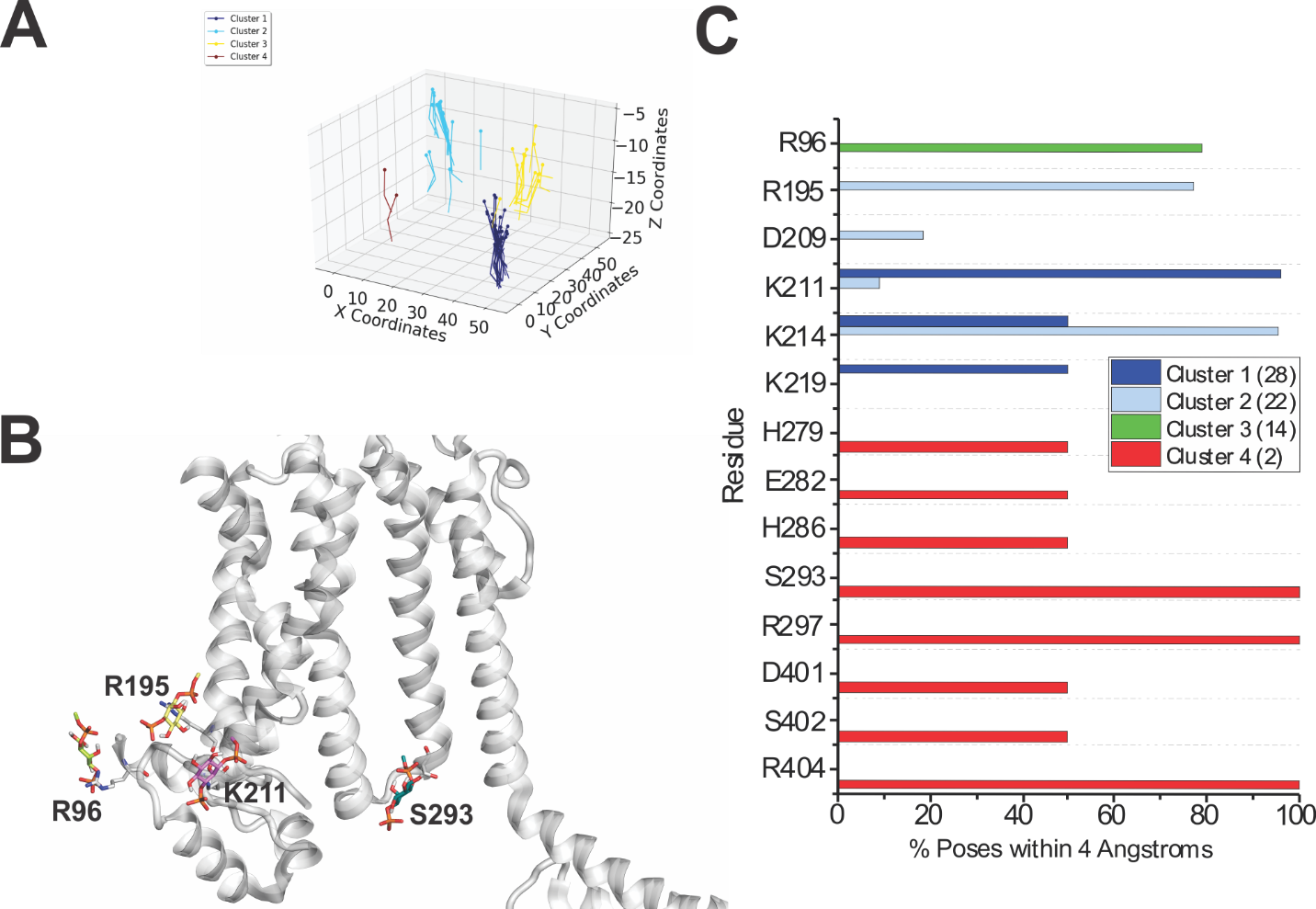
**

**Supp. Fig. S2**. **Docking of PI(3)P to HCN1 channels with VSDs in the up (closed) conformation. (A)** The results from 500 attempts to dock PI(3)P headgroups to HCN1 (PDB: 5U6O). Poses were accepted based on orientation and clusters were identified in an automated manner using MultiCluster software previously described (Tanguay et al., 2019). **(B)** Binding sites of the most representative poses for each cluster **(C)** The frequency of residues within 4 Å of the ligand for each pose was assessed and is indicated as a percentage of the accepted poses.


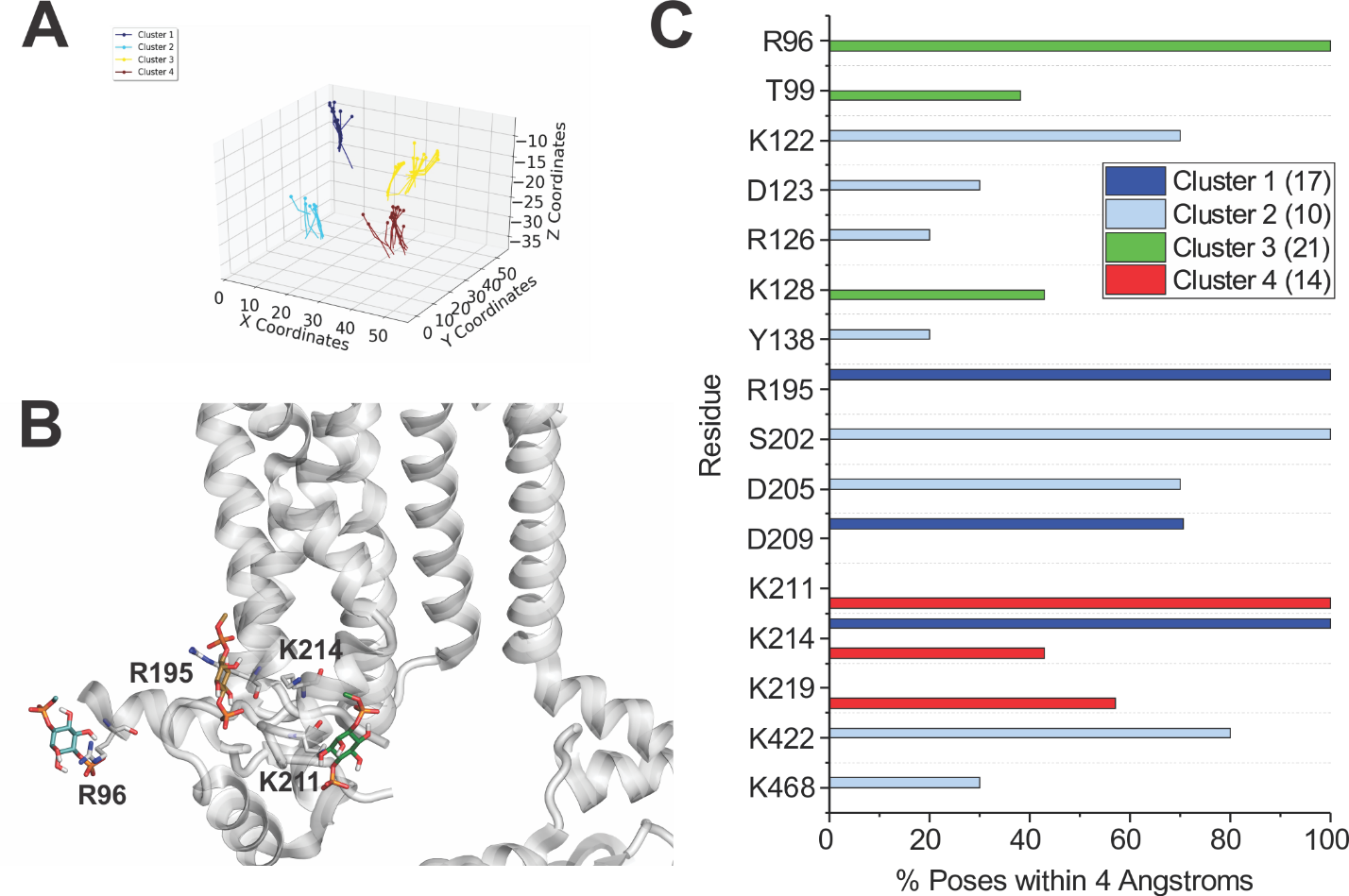


**Supp. Fig. S3**. **Docking of PI(4)P to HCN1 channels with VSDs in the up (closed) conformation. (A)** The results from 500 attempts to dock PI(4)P headgroups to HCN1 (PDB: 5U6O). Poses were accepted based on orientation and clusters were identified in an automated manner using MultiCluster software previously described (Tanguay et al., 2019). **(B)** Binding sites of the most representative poses for each cluster **(C)** The frequency of residues within 4 Å of the ligand for each pose was assessed and is indicated as a percentage of the accepted poses.


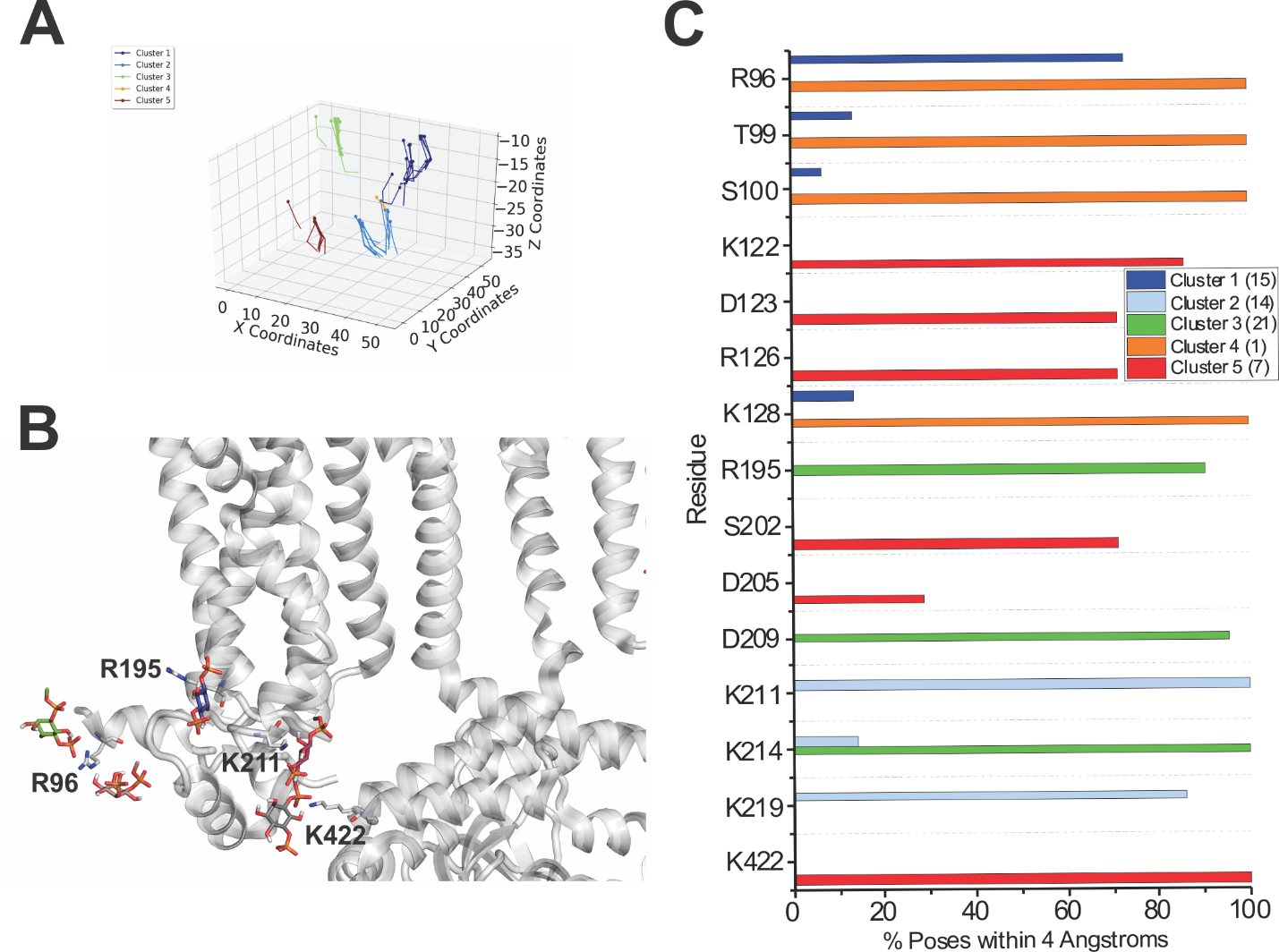


**Supp. Fig. S4**. **Docking of PI(5)P to HCN1 channels with VSDs in the up (closed) conformation. (A)** The results from 500 attempts to dock PI(5)P headgroups to HCN1 (PDB: 5U6O). Poses were accepted based on orientation and clusters were identified in an automated manner using MultiCluster software previously described (Tanguay et al., 2019). **(B)** Binding sites of the most representative poses for each cluster **(C)** The frequency of residues within 4 Å of the ligand for each pose was assessed and is indicated as a percentage of the accepted poses.


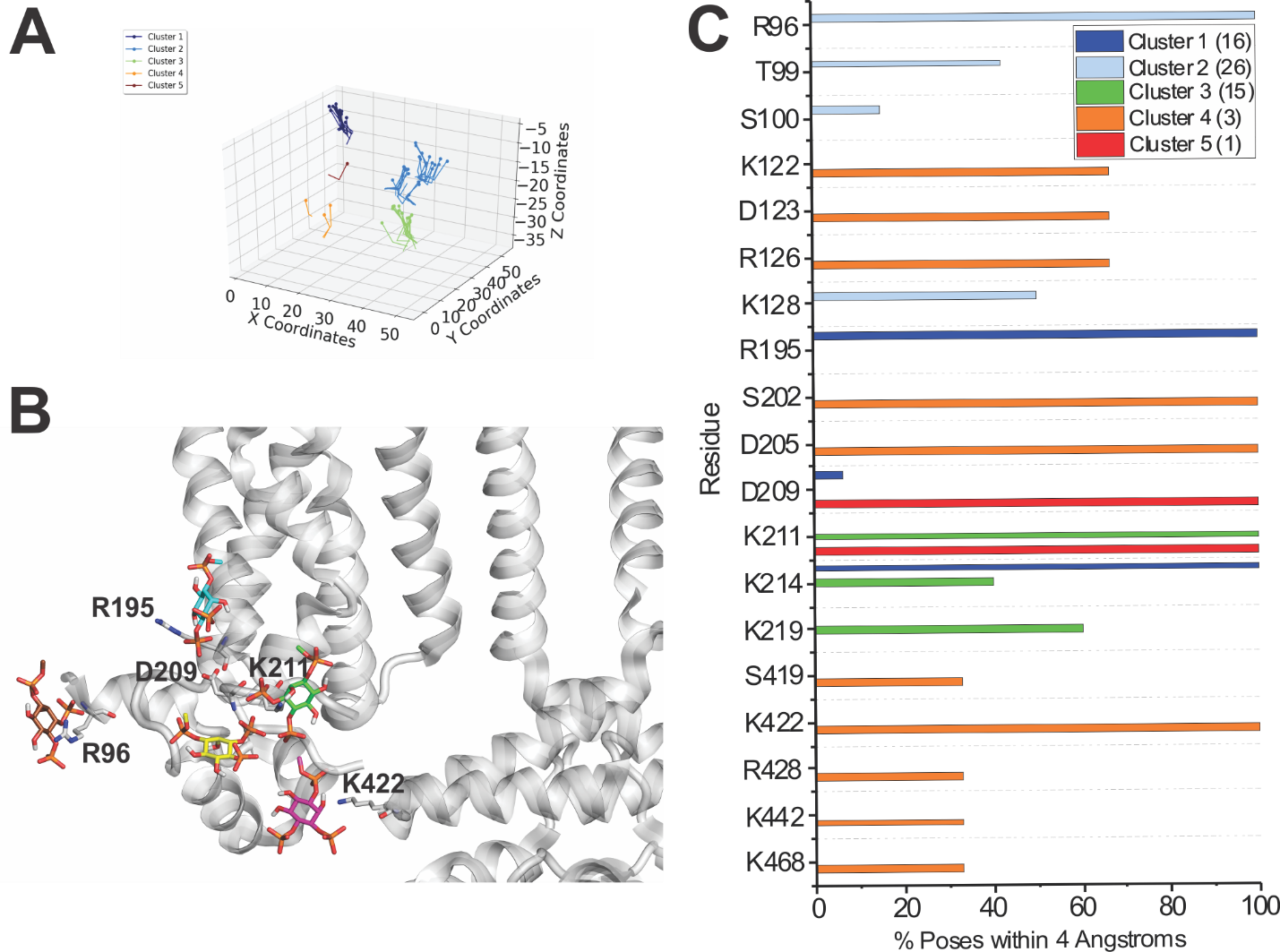


**Supp. Fig. S5**. **Docking of PI(3,4)P_2_ to HCN1 channels with VSDs in the up (closed) conformation. (A)** The results from 500 attempts to dock PI(3,4)P_2_ headgroups to HCN1 (PDB: 5U6O). Poses were accepted based on orientation and clusters were identified in an automated manner using MultiCluster software previously described (Tanguay et al., 2019). **(B)** Binding sites of the most representative poses for each cluster **(C)** The frequency of residues within 4 Å of the ligand for each pose was assessed and is indicated as a percentage of the accepted poses.


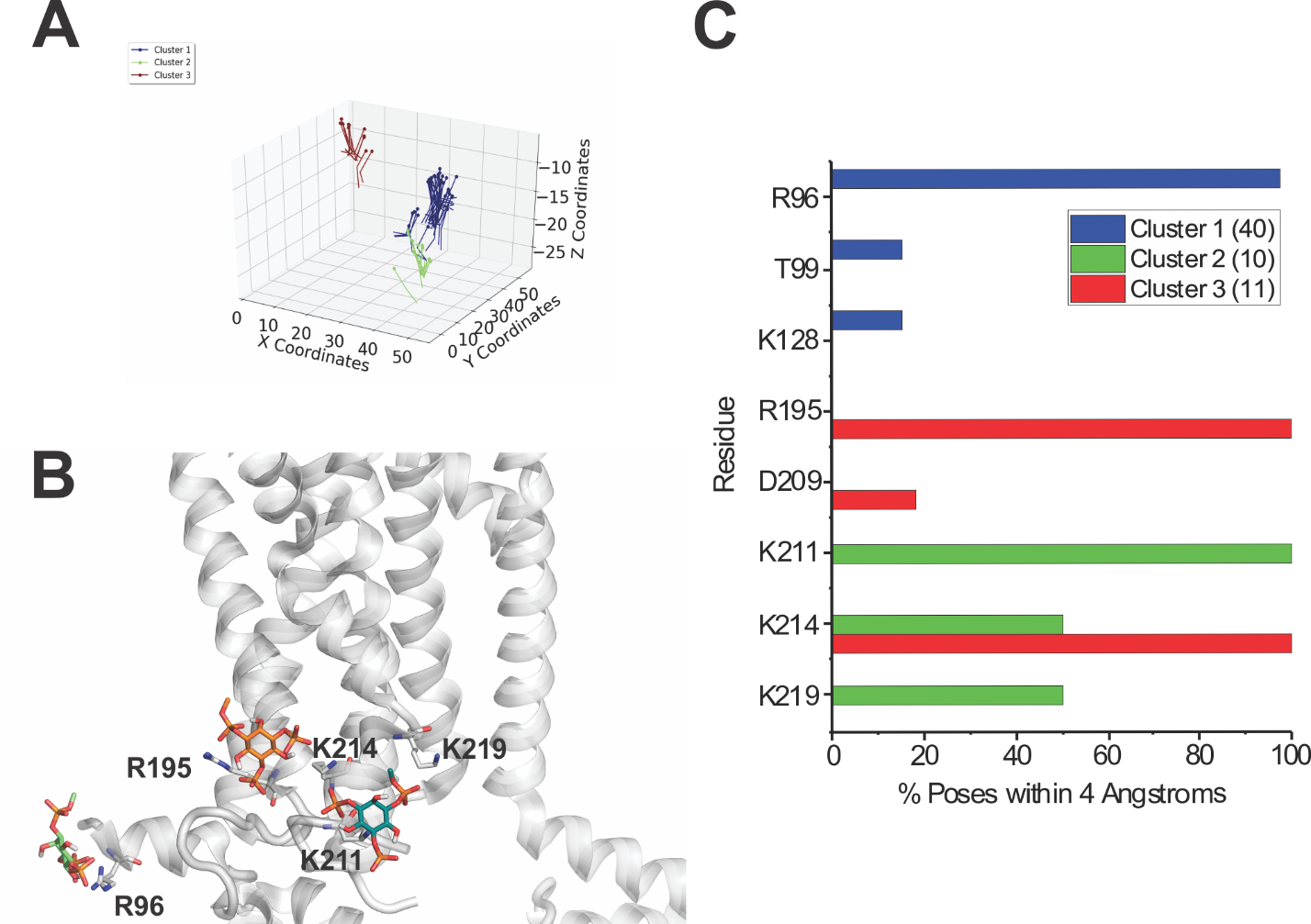


**Supp. Fig. S6**. **Docking of PI(3,5)P_2_ to HCN1 channels with VSDs in the up (closed) conformation. (A)** The results from 500 attempts to dock PI(3,5)P_2_ headgroups to HCN1 (PDB: 5U6O). Poses were accepted based on orientation and clusters were identified in an automated manner using MultiCluster software previously described (Tanguay et al., 2019). **(B)** Binding sites of the most representative poses for each cluster **(C)** The frequency of residues within 4 Å of the ligand for each pose was assessed and is indicated as a percentage of the accepted poses.

**
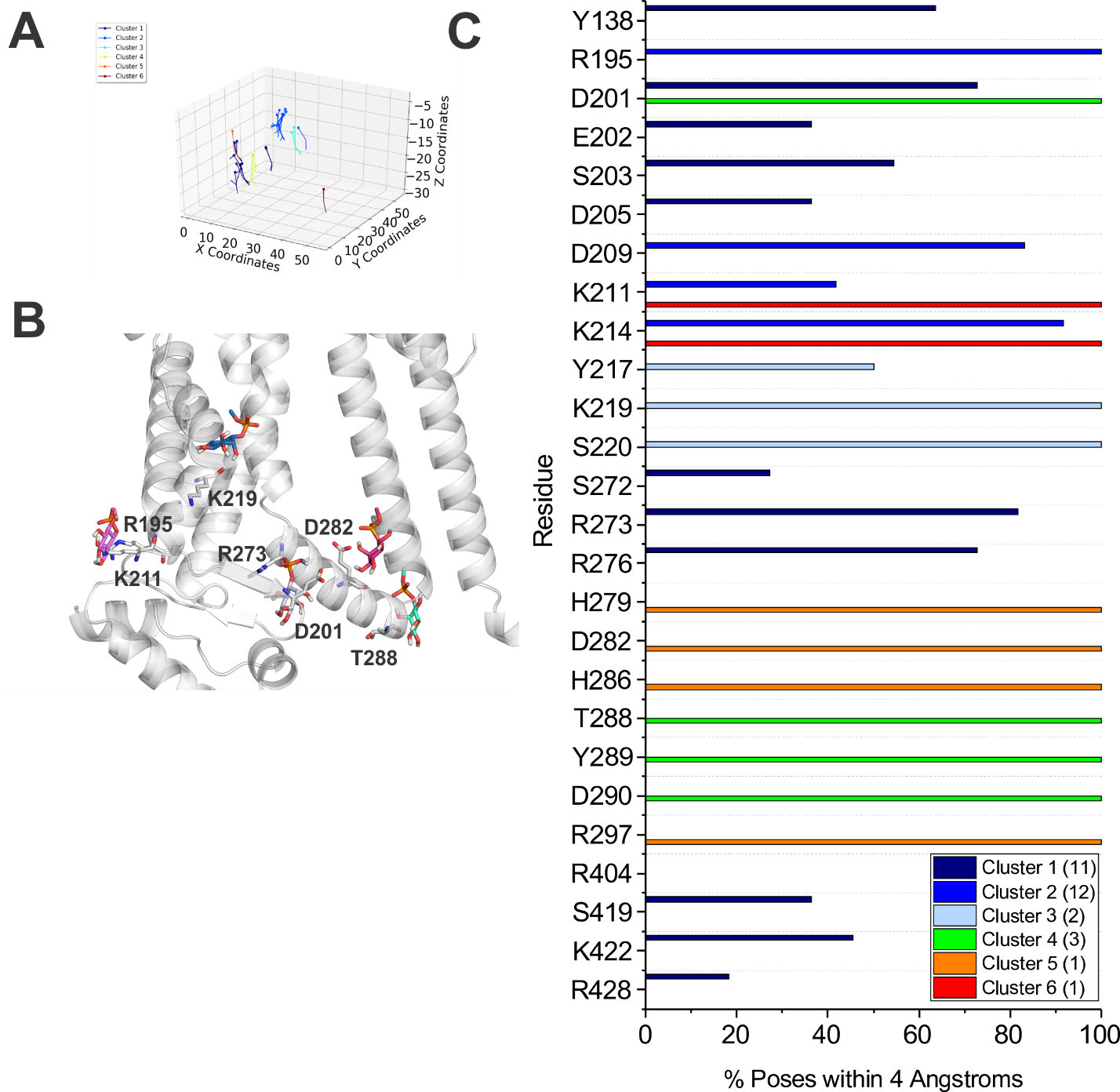
**

**Supp. Fig. S7**. **Docking of PI to HCN1 channels with VSDs in the down (activated) conformation. (A)** The results from 500 attempts to dock PI headgroups to HCN1 (PDB: 6UQF). Poses were accepted based on orientation and clusters were identified in an automated manner using MultiCluster software previously described (Tanguay et al., 2019). **(B)** Binding sites of the most representative poses for each cluster **(C)** The frequency of residues within 4 Å of the ligand for each pose was assessed and is indicated as a percentage of the accepted poses.

**
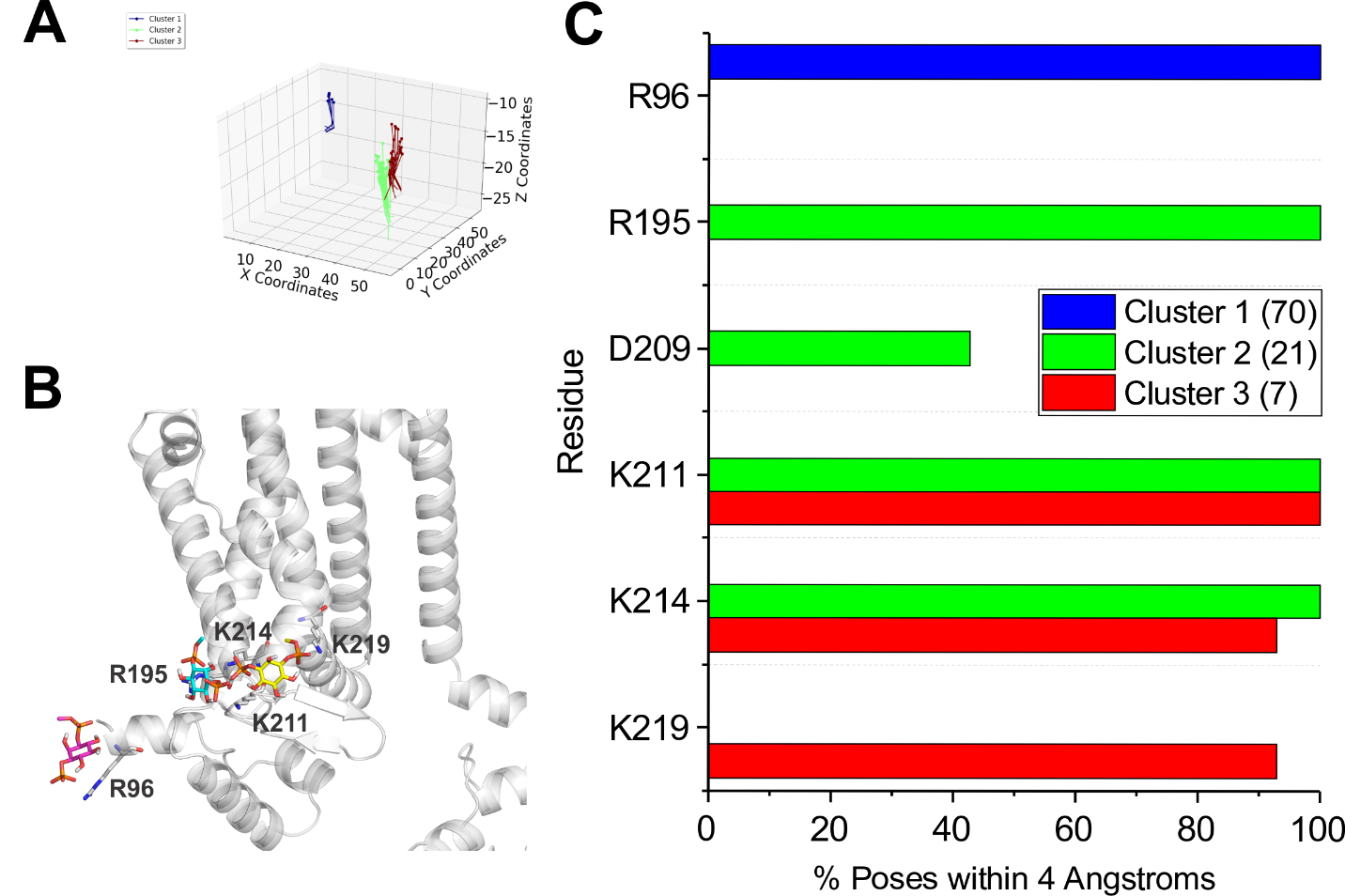
**

**Supp. Fig. S8**. **Docking of PI(3)P to HCN1 channels with VSDs in the down (activated) conformation. (A)** The results from 500 attempts to dock PI(3)P headgroups to HCN1 (PDB: 6UQF). Poses were accepted based on orientation and clusters were identified in an automated manner using MultiCluster software previously described (Tanguay et al., 2019). **(B)** Binding sites of the most representative poses for each cluster **(C)** The frequency of residues within 4 Å of the ligand for each pose was assessed and is indicated as a percentage of the accepted poses.


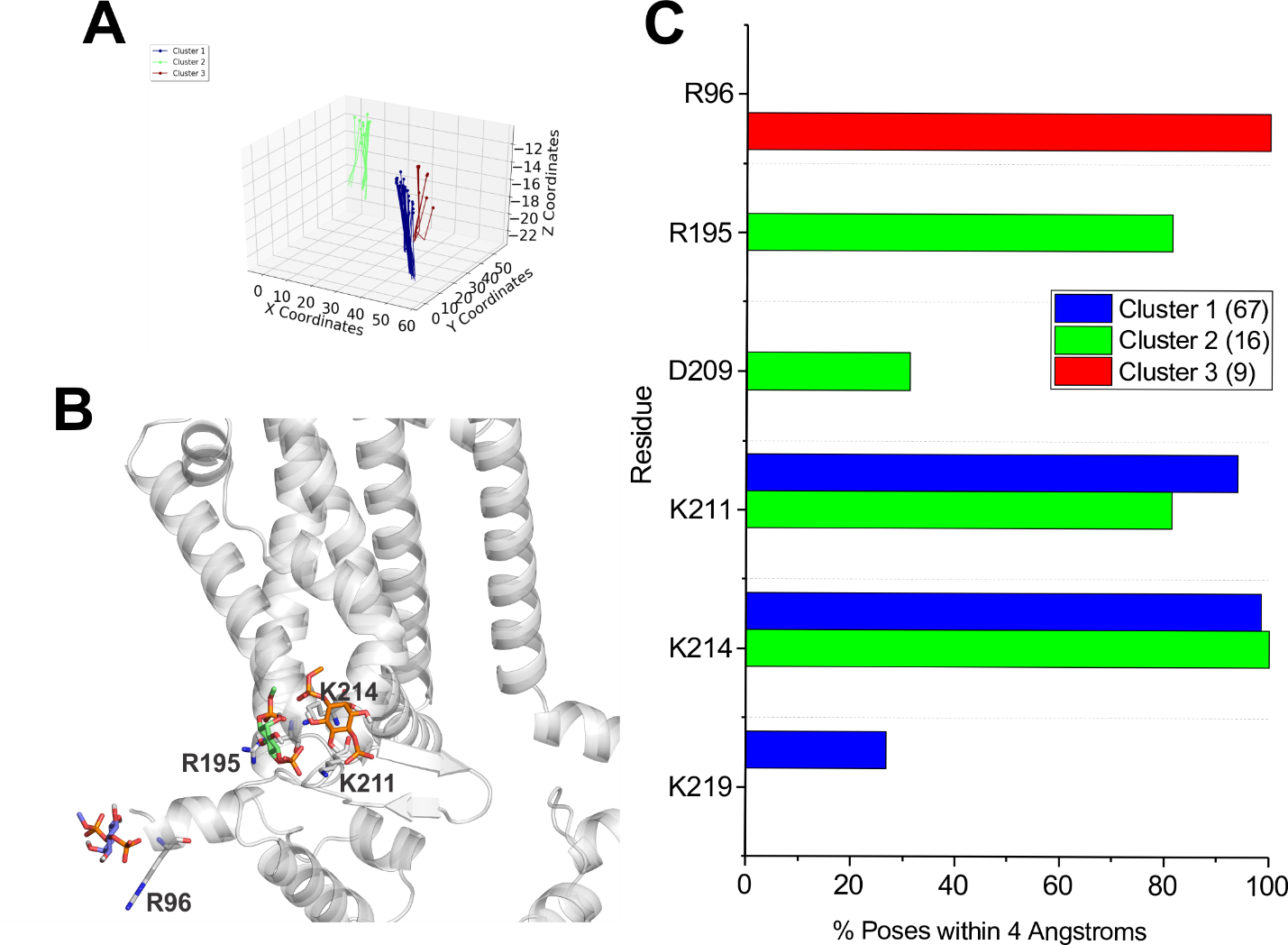


**Supp. Fig. S9**. **Docking of PI(4)P to HCN1 channels with VSDs in the down (activated) conformation. (A)** The results from 500 attempts to dock PI(4)P headgroups to HCN1 (PDB: 6UQF). Poses were accepted based on orientation and clusters were identified in an automated manner using MultiCluster software previously described (Tanguay et al., 2019). **(B)** Binding sites of the most representative poses for each cluster **(C)** The frequency of residues within 4 Å of the ligand for each pose was assessed and is indicated as a percentage of the accepted poses.


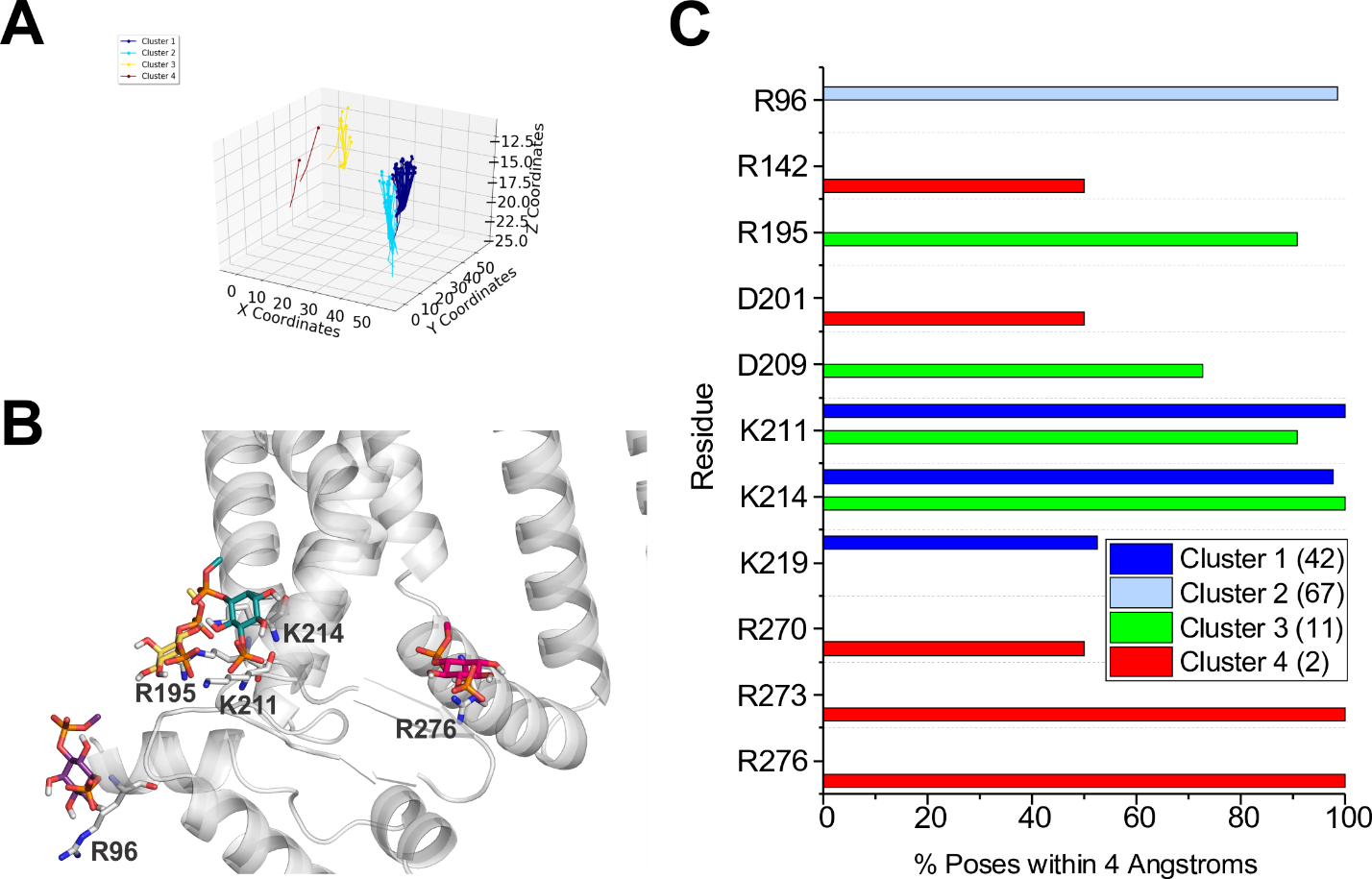


**Supp. Fig. S10**. **Docking of PI(5)P to HCN1 channels with VSDs in the down (activated) conformation. (A)** The results from 500 attempts to dock PI(5)P headgroups to HCN1 (PDB: 6UQF). Poses were accepted based on orientation and clusters were identified in an automated manner using MultiCluster software previously described (Tanguay et al., 2019). **(B)** Binding sites of the most representative poses for each cluster **(C)** The frequency of residues within 4 Å of the ligand for each pose was assessed and is indicated as a percentage of the accepted poses.


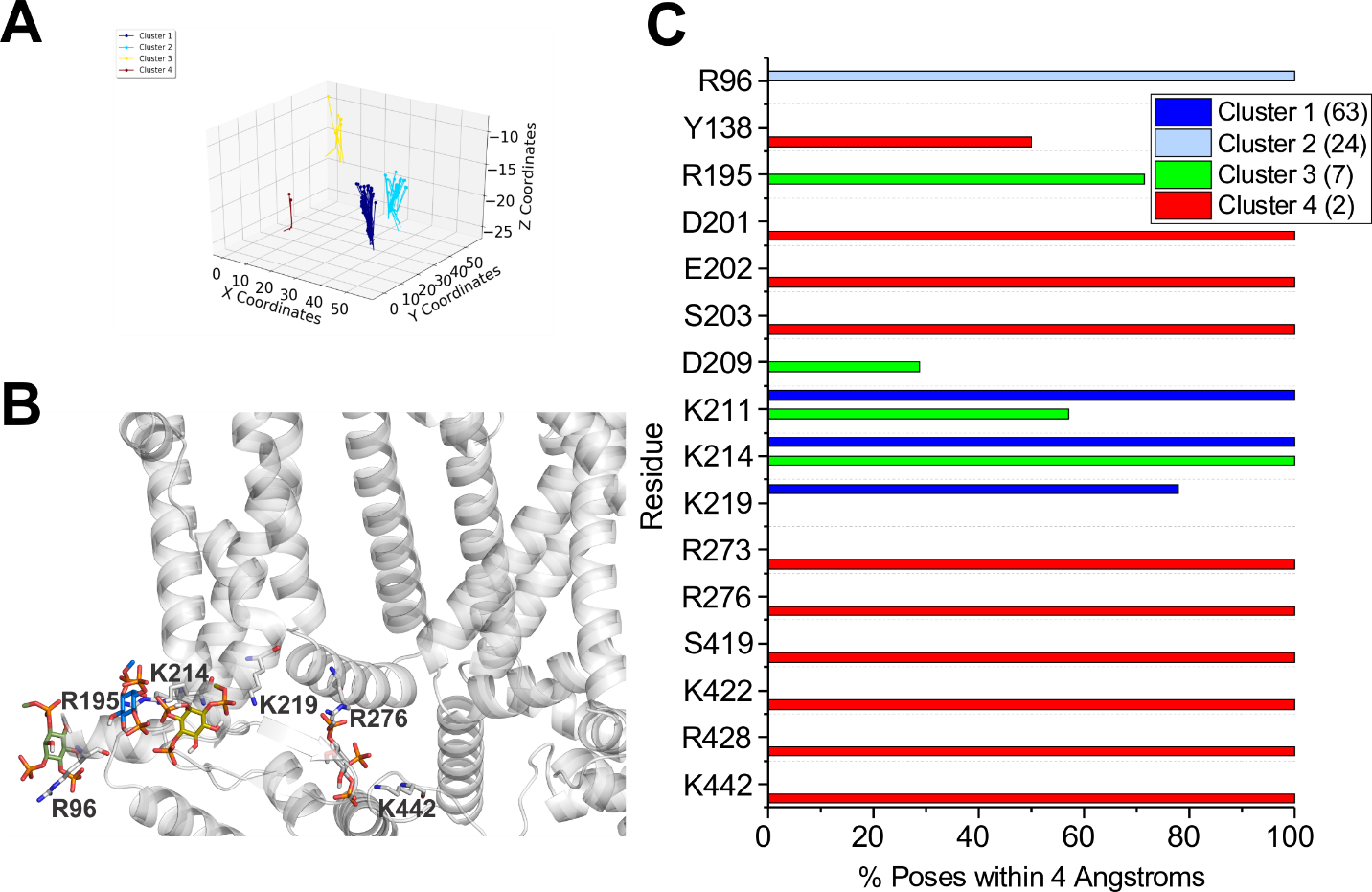


**Supp. Fig. S11**. **Docking of PI(3,4)P_2_ to HCN1 channels with VSDs in the down (activated) conformation. (A)** The results from 500 attempts to dock PI(3,4)P_2_ headgroups to HCN1 (PDB: 6UQF). Poses were accepted based on orientation and clusters were identified in an automated manner using MultiCluster software previously described (Tanguay et al., 2019). **(B)** Binding sites of the most representative poses for each cluster **(C)** The frequency of residues within 4 Å of the ligand for each pose was assessed and is indicated as a percentage of the accepted poses.


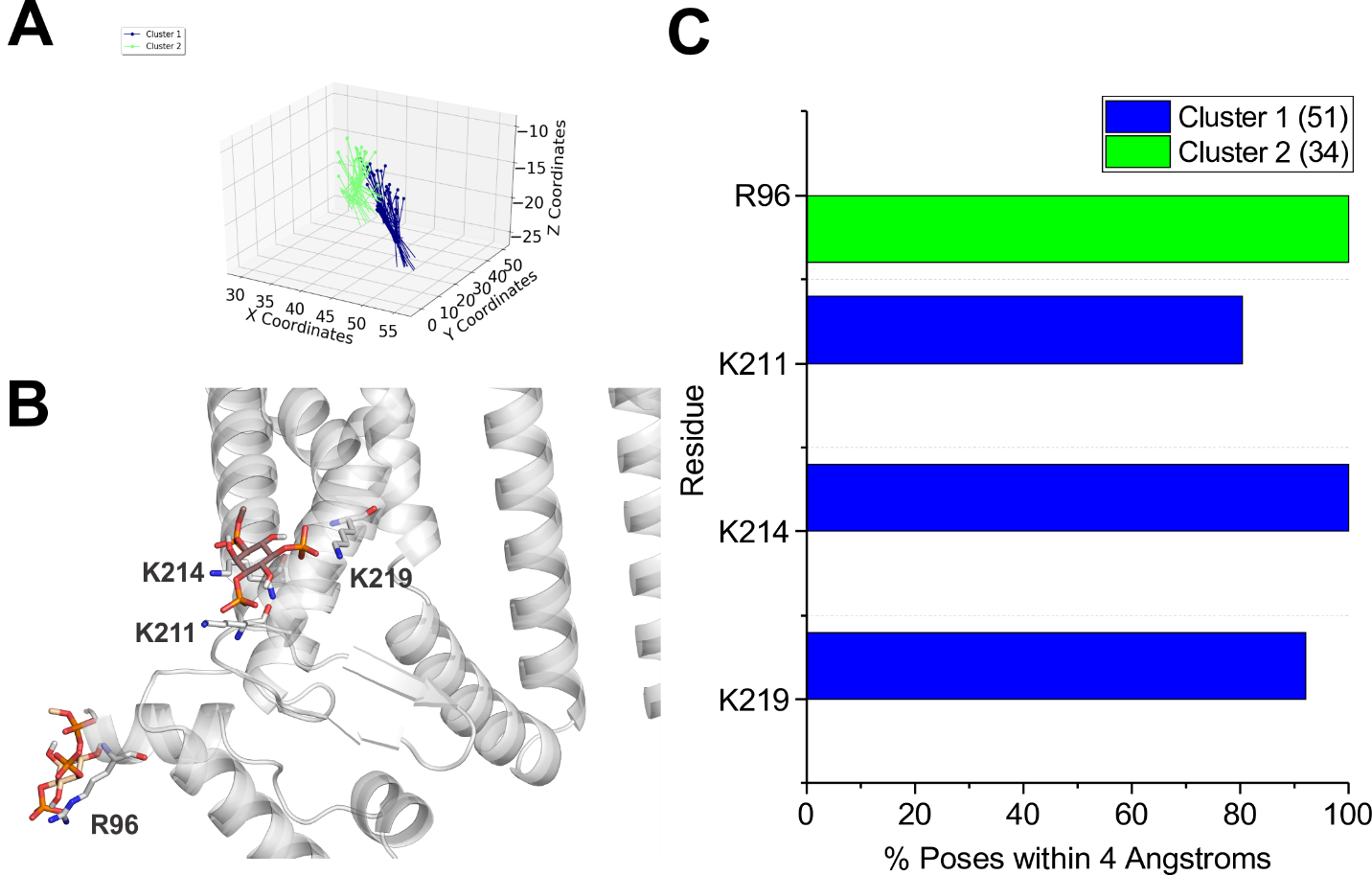


**Supp. Fig. S12**. **Docking of PI(3,5)P_2_ to HCN1 channels with VSDs in the down (activated) conformation. (A)** The results from 500 attempts to dock PI(3,5)P_2_ headgroups to HCN1 (PDB: 6UQF). Poses were accepted based on orientation and clusters were identified in an automated manner using MultiCluster software previously described (Tanguay et al., 2019). **(B)** Binding sites of the most representative poses for each cluster **(C)** The frequency of residues within 4 Å of the ligand for each pose was assessed and is indicated as a percentage of the accepted poses.

**
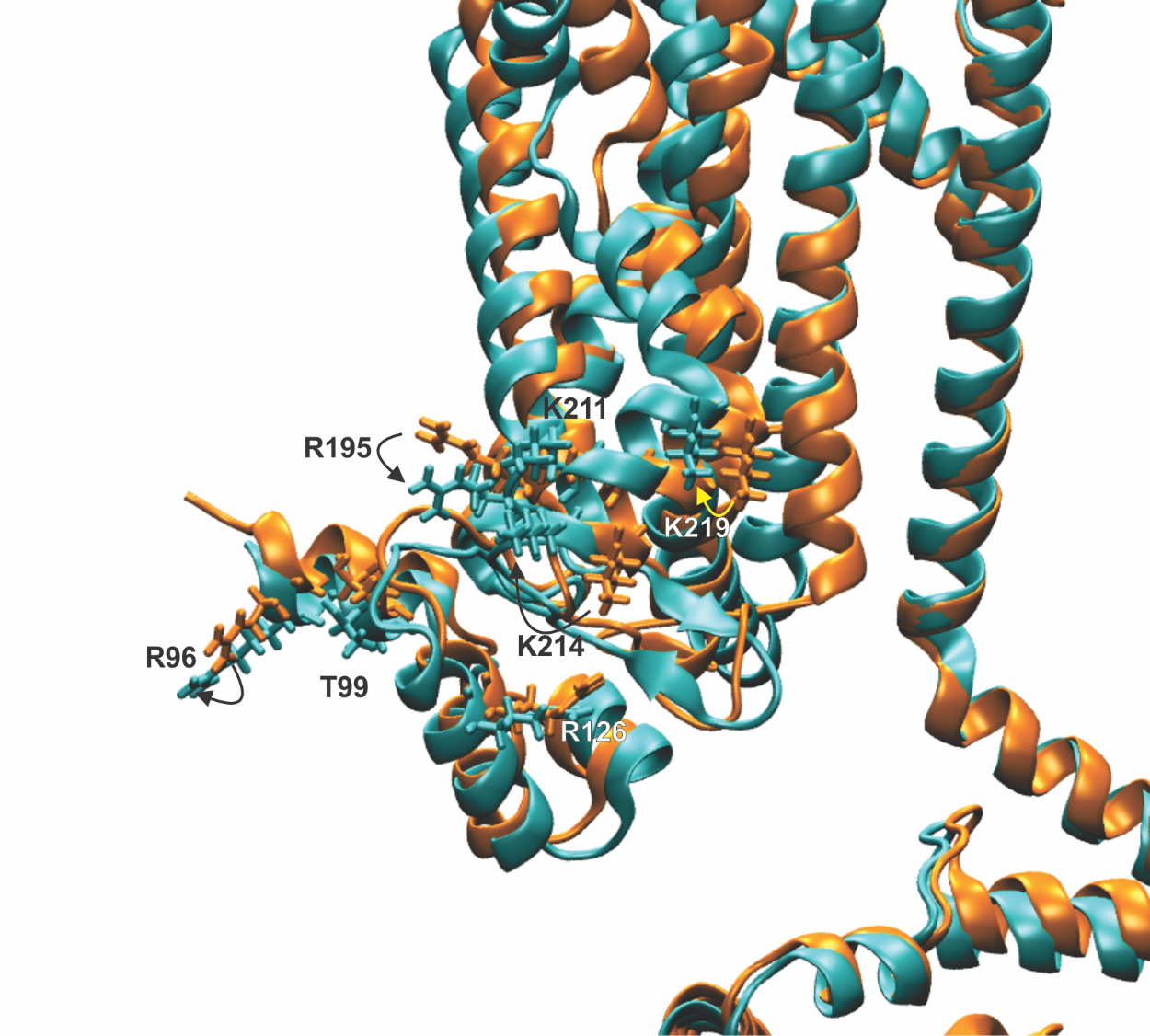
**

**Supp. Fig. S13**. **Overlay of an HCN1 subunits with VSDs in the up and down conformations.** HCN1 channels with their VSD in the up (PDB: 5U6O; orange) or down (PDB: 6UQF; cyan) conformation by superimposition of the S6, selectivity filter and pore-helix. Downward movement of the VSD rotates the HCNa helix of the HCN domain positioning R96, T99 and S100 further away from the plane of the membrane. Furthermore, movement of the S2 and S3 positions K211 and K214 closer to R195 in the down state compared to the up state. It is conceivable that these changes may contribute to state dependent binding of PIPs to HCN channels.


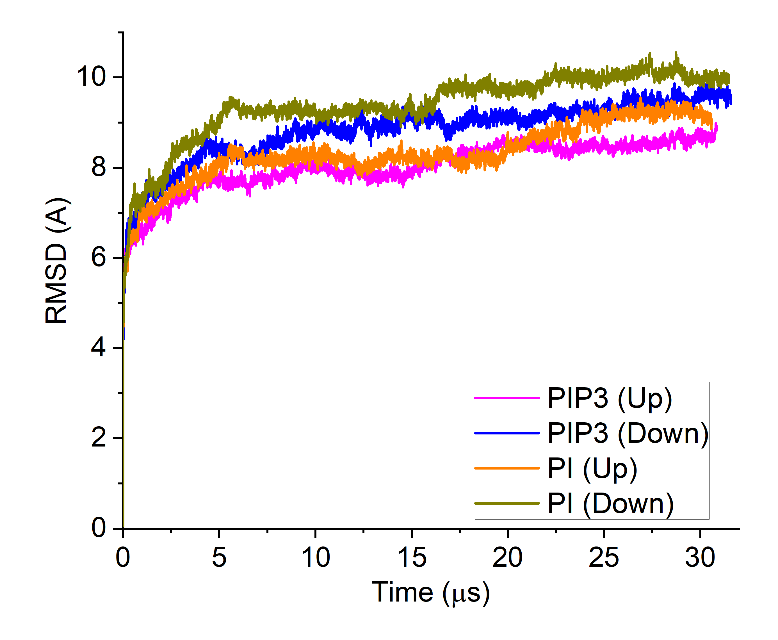


**Supp. Fig. S14**. **RMSD of HCN1 proteins during coarse-grained simulations.** RMSD of protein residues were calculated over each trajectory calculated against the first frame of that system following equilibration.
